# The Relationship Between Clinical Phenotypes and Global Initiative for Chronic Obstructive Lung Disease (GOLD) Stages/Groups in Patients With Chronic Obstructive Pulmonary Disease

**DOI:** 10.7759/cureus.32116

**Published:** 2022-12-01

**Authors:** Anuj Kumar Pandey, Ajay Kumar Verma, Arpita Singh, Surya Kant, Rakesh Kumar Dixit, Shyam Chand Chaudhary, Jyoti Bajpai, Ved Prakash, Umesh Pratap Verma

**Affiliations:** 1 Respiratory Medicine, King George's Medical University, Lucknow, IND; 2 Pharmacology, Dr Ram Manohar Lohia Institute of Medical Sciences, Lucknow, IND; 3 Pharmacology and Therapeutics, King George's Medical University, Lucknow, IND; 4 Internal Medicine, King George's Medical University, Lucknow, IND; 5 Pulmonary and Critical Care Medicine, King George's Medical University, Lucknow, IND; 6 Periodontology, King George's Medical University, Lucknow, IND

**Keywords:** risks for hospitalization, mmrc dyspnea scale, copd assessment test, management, copd, spirometry, exacerbation, symptoms, airflow limitation, abcd assessment tool

## Abstract

Background

Chronic obstructive pulmonary disease (COPD) cannot be properly characterised by a single metric, forced expiratory volume in the first second (FEV1), due to its complexity and heterogeneity. The GOLD 2017 report contained the ABCD evaluation method to measure airflow limitation, symptoms, and/or exacerbation risk.

Objective

The purpose of this study was to explore the relationship between clinical characteristics and GOLD groups or stages in patients with COPD.

Methods

This cross-sectional observational study was conducted at the department of respiratory medicine, King George’s Medical University, Lucknow, Uttar Pradesh, India, between 2019 and 2022. Here, stable COPD patients' demographics, clinical characteristics, and the number of exacerbations were compared between the groups following the GOLD 2022 report. An unpaired t-test with Welch's correction, chi-square test, Fisher's exact test, one-way ANOVA, and Kruskal-Wallis test were used for statistical significance.

Results

In this study, 349 stable COPD patients (256 males and 93 females) were selected. The GOLD 2017 categorization placed 78 (22.4%) patients in group A, 158 (45.3%) in B, 44 (12.6%) in C, and 69 (19.8%) in D. Further, we used GOLD 2017 to classify COPD patients into 16 subgroups (1A-4D). FEV1 (% predicted) decreased across groups A to D (p<0.0001). Groups C and D had a longer duration of illness, higher COPD assessment test (CAT) score, higher Modified Medical Research Council (mMRC) dyspnea scale, longer exacerbation history, and more COPD hospitalizations in the previous year than groups A and B. More symptomatic patients (B and D) exhibited lower FEV1 (% predicted) and more severe airflow limitation than less symptomatic patients (A and C) (p=0.0002). Symptomatic individuals exhibited higher CAT and mMRC dyspnea scores (p<0.0001). Groups C and D comprised older patients and those with longer disease duration, higher mMRC dyspnea scale and CAT, lower FEV1, and more severe airflow limitation (A and B).

Conclusion

The present study demonstrates the distribution of COPD patients' clinical phenotypes in an Indian population. We conclude that the combined COPD assessment according to the GOLD 2022 guideline provides a better understanding of COPD.

## Introduction

An estimated 300 million people throughout the world are living with chronic obstructive pulmonary disease (COPD), a progressive lung disorder that is chronic in nature and is characterised by respiratory symptoms linked to persistent airflow limitation [1]. The severity of COPD has historically only been determined by the forced expiratory volume in 1 second (FEV1), as determined by spirometry [2-4]. More than 50 years ago, various authors used FEV1 to categorise the severity of COPD in studies of the disease's natural history, and since then, spirometry-based severity systems have been known to be reliable predictors of survival in COPD-related studies worldwide [2-5]. The Global Initiative for Chronic Obstructive Lung Disease (GOLD) classification method has been used to categorise COPD since 2007. The GOLD 2007 classification [6] was based only on FEV1 thresholds and expected normal values. Patients had to have an FEV1/ forced vital capacity (FVC) ratio of less than 0.70. Patients are classified as stage 1 (mild), stage 2 (moderate), stage 3 (severe), and stage 4 (very severe).

Traditional spirometry-based COPD severity indices measure just airflow, ignoring persistent symptoms and comorbidities. In COPD, clinical manifestations, imaging reports, lung function tests, therapeutic response, and patient survival are all variables [5-8]. The problem of over-diagnosis of COPD occurs when the standard definition of obstruction as an FEV1/FVC ratio of less than 0.70 is applied to older populations, highlighting the limits of using spirometry to define COPD [4, 9]. There is now general agreement that FEV1 measurement alone should not be used for the best diagnosis, evaluation, and management of individuals with COPD since it does not accurately reflect the complexity of the disease. Hurst et al. demonstrated that pulmonary function is not the best predictor of having frequent exacerbations, which are defined as two or more exacerbations per year [5].

As a result, the 2011 GOLD revision incorporated the assessment of symptoms, severity of airflow limitation, and history of exacerbations into severity scores [10]. By combining the symptom assessment with the patient's spirometric classification and/or risk of exacerbations, we were able to learn more about how COPD affects each person. The GOLD 2011 classification [10-11] was based on an ABCD assessment. The GOLD 2017 report updates the GOLD 2011 report. The GOLD 2017 classification [12] distinguishes between the ABCD classes determined by symptoms and exacerbations in the 2011 classification and the spirometric staging (1-4) in the 2007 classification. In the present study, a classification system that was based on a combination of spirometry, symptoms, and/or exacerbations was used to divide participants into a total of 16 subgroups (1A to 4D). In this study, we sought to find out how the different GOLD definitions affect the demographics as well as clinical characteristics of COPD patients and performed a thorough assessment of COPD patients using the GOLD 2017 classification.

## Materials and methods

Study design and patients

**Figure 1:**
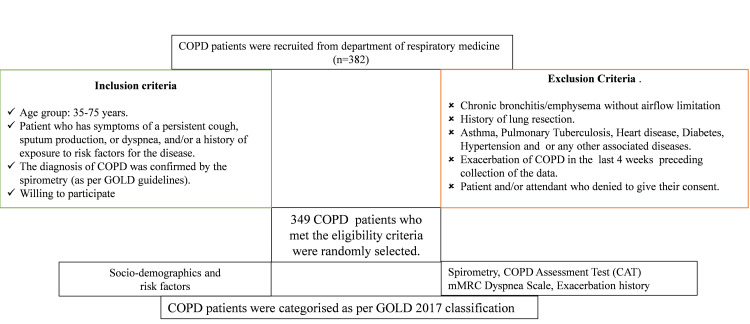
Study CONSORT flow diagram Abbreviations: COPD, chronic obstructive pulmonary disease; GOLD, Global Initiative for Chronic Obstructive Lung Disease, mMRC Dyspnea Scale, Modified Medical Research Council Dyspnea Scale; CAT score, COPD assessment test score

This prospective observational cross-sectional study was conducted at the respiratory medicine department of a tertiary care centre. Patients visiting the outpatient department were recruited. COPD was suspected in patients with chronic cough, breathlessness, and a history of exposure to disease risk factors. The study CONSORT with inclusion and exclusion criteria was depicted in Figure 1. Data related to demographics, smoking history, medical history, clinical symptoms, body mass index (BMI), COPD assessment test (CAT), Modified Medical Research Council (mMRC) Dyspnea Scale, and exacerbation history were documented. The GOLD classification system (GOLD 2007, GOLD 2011, and GOLD 2017) is compiled in Table 1.

**Table 1:** Different GOLD classifications for COPD Abbrevations: FEV1, forced expiratory volume in the first second; FVC, forced vital capacity; mMRC Dyspnea Scale, Modified Medical Research Council Dyspnea Scale; CAT score, COPD assessment test score

GOLD 2007 [6,10-11]	GOLD 2011 [10,11-13]	GOLD 2017 [8,10-12]
Based on FEV1thresholds compared with predicted normal values, with FEV1/FVC less than 0·70.	Based on symptoms, the severity of airflow limitation, and history of exacerbations: ABCD assessment tool.	Separated spirometric staging (1–4) from the ABCD groups.
Stage 1 (mild): FEV1 ≥80% predicted.	Group A: mMRC 0–1, CAT <10, GOLD 1 or 2, Exacerbation history 0–1.	Group A: mMRC 0–1, CAT <10, Exacerbation history 0 or 1 (not leading to hospital admission).
Stage 2 (moderate): 50% ≤ FEV1​​​​​​​, <80% predicted	Group B: mMRC ≥ 2, CAT ≥10, GOLD 1 or 2, Exacerbation history 0–1.	Group B: mMRC ≥ 2, CAT ≥10, Exacerbation history 0 or 1 (not leading to hospital admission).
Stage 3 (severe): 30% ≤ FEV1​​​​​​​, <50% predicted	Group C: mMRC 0–1, CAT <10, GOLD 3 or 4, Exacerbation history 0–1.	Group C: mMRC 0–1, CAT <10, Exacerbation history ≥2 or ≥1 (leading to hospital admission).
Stage 4 (very severe): FEV1 ≤30% predicted or FEV1 ≤50% predicted with chronic respiratory failure.	Group D: mMRC ≥ 2, CAT ≥10, GOLD 3 or 4, Exacerbation history 0–1.	Group D: mMRC ≥ 2, CAT ≥10 Exacerbation history ≥2 or ≥1 (leading to hospital admission).

Lung function test

Spirometry was performed by a trained technician with the Spiropalm spirometer (Cosmed Srl, Rome, Italy) in suspected cases [13]. GOLD guidelines confirmed and characterised COPD diagnostic criteria as follows: GOLD-1, predicted FEV1​​​​​​​ percentage was ≥80%; GOLD-2, predicted FEV1​​​​​​​ percentage was ≥50% but <80%; GOLD-3, predicted FEV1​​​​​​​ percentage was ≥30% but <50%; and GOLD-4, predicted FEV1​​​​​​​_ _percentage was <30% [13]. 

Clinical measurements

The COPD assessment test (CAT) is an eight-item, unidimensional test used to assess COPD-related health status [14]. It was designed to be used everywhere, and there are approved translations available in a variety of languages. The CAT score varies from 0-40, has a strong correlation with the SGRQ, and has been thoroughly researched in various studies. Dyspnea was evaluated using the mMRC Dyspnea Scale. The administration of antibiotics and/or systemic corticosteroids for the treatment of worsening respiratory symptoms was regarded as an exacerbation when there was insufficient evidence to indicate an alternative diagnosis.

The ABCD assessment tool

In the ABCD classification system, the mMRC dyspnea scale or the CAT is used to assess symptom burden, and the GOLD grades of airflow limitation or exacerbation history should be used to assess exacerbation risk. The ABCD categorization is based on a 2x2 cell table labelled A, B, C, and D. The symptom burden is divided into two categories: low (A and C) and high (B and D), with airflow limitation and/or exacerbation history distinguishing A from C and B from D. The ABCD grouping was conducted following the GOLD 2007, GOLD 2011, and GOLD 2017 (Table 1) classification systems.

Statistical analysis

All data were presented as the mean, standard deviation (SD), number (n), and percentage (%). The characteristics of the COPD in the GOLD groups (A, B, C, D) were analysed using one-way analysis of variance (ANOVA), the Kruskal-Wallis test, and/or the chi-square test. Further, differences between the two groups were assessed using the unpaired t-test with Welch's correction, the chi-square test, and/or Fisher's exact test. GraphPad Prism 6 (GraphPad Software Inc., San Diego, CA, USA) was used to sum up the data. The following p-values were considered: p>0.05, p≤0.05, p≤0.01, and p≤0.001.

Ethics statement

The study was approved by the institutional ethics committee (IEC) of King George’s Medical University, Lucknow, Uttar Pradesh, India. Before enrolment, all participants provided written informed consent. In addition to this, the study was done according to the Declaration of Helsinki.

## Results

Study subject distribution according to GOLD 2007, GOLD 2011, and GOLD 2017 classifications

**Figure 2:**
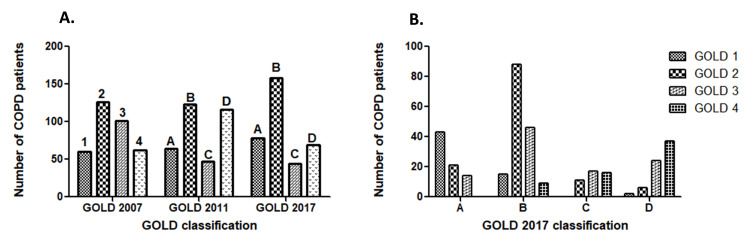
Distribution of the studied COPD patients according to GOLD 2007, GOLD 2011, GOLD 2017 classification Abbreviations: COPD, chronic obstructive pulmonary disease; GOLD, Global Initiative for Chronic Obstructive Lung Disease.

In this study, 349 stable COPD patients (256 males and 93 females, mean age 60.7 (11.2)) were selected. Patients were distributed (GOLD-1234 or GOLD-ABCD) according to GOLD 2007, GOLD 2011, and GOLD 2017 as stated in Figure 2. According to the GOLD 2007 classification, 60 patients were in Grade 1, 126 were in Grade 2, 101 were in Grade 3, and 62 were in Grade 4 (Figure 2). According to the GOLD 2011 (Figure 2A), 64 patients were placed in group A, 122 patients were placed in group B, 47 patients were placed in group C, and 116 patients were placed in group D. According to the GOLD 2017 classification, 78 (22.4%) patients were assigned to group A, 158 (45.3%) to group B, 44 (12.6%) to group C, and 69 (19.8%) to group D (Figure 2).

According to the GOLD 2011 report, patients who were previously considered to be at low risk were reclassified as being at high risk in the GOLD 2017 updates (Figure 2A). In addition, some individuals who were classified as having a high risk according to the GOLD 2011 have been reclassified as having a low risk owing to the GOLD 2017 updates (Figure 2B). As per the GOLD 2017 reports, the majority of patients were classified as group B (Figure 2B). We classified COPD patients according to the GOLD 2017 classification and further divided them into 16 sub-groups (1A to 4D) (Figures 2A-B, Table 2).

**Table 2:** Distribution of studied COPD patients according to GOLD 2017 classification Note: Data are presented in n (%). Abbreviations: n, number of COPD patients; %, percent; GOLD, Global Initiative for Chronic Obstructive Lung Disease; COPD, chronic obstructive pulmonary disease

	Group A	Group B	Group C	Group D	Total
Total	78 (22.4%)	158 (45.3%)	44 (12.6%)	69 (19.8%)	349 (100%)
GOLD 1	43 (55.1%)	15 (9.5%)	0 (0%)	2 (2.9%)	60 (17.2%)
GOLD 2	21 (26.9%)	88 (55.7%)	11 (25%)	6 (8.7%)	126 (36.1%)
GOLD 3	14 (17.9%)	46 (29.1%)	17 (38.6%)	24 (34.8%)	101 (28.9%)
GOLD 4	0 (0%)	9 (5.7%)	16 (36.4%)	37 (53.6%)	62 (17.8%)

Baseline characteristics of COPD patients based on the 2017 GOLD classification

In Table 3, the baseline characteristics of patients with COPD following the GOLD 2017 updates are presented. The group D patients were older than the A, B, and/or C groups. The overall value (mean, SD) of FEV1 (% predicted) was 54.6 (19.6), while across the groups A to D it was significantly decreased (A, 70.6 (15.3); B, 59 (15.2); C, 42.2 (15); D, 36.3 (14.5); one-way ANOVA, p<0.0001, Table 3). The duration of illness, CAT, mMRC dyspnea Scale, exacerbation history, and hospitalisation due to COPD exacerbation in the previous year were higher in groups (C and D) than in groups (A and B). Other than this, there was no significant difference in smoking history, biomass fuel exposure, residence, and/or marital status between groups A, B, C, and/or D.

**Table 3:** Baseline characteristics of patients with COPD according to GOLD 2017 classification Notes: Data are expressed in mean (SD), and n(%). ^$^chi-square test, ^#^one-way ANOVA, ^ns^p>0.05, *p≤0.05, **p≤0.01, ***p≤0.001 Abbreviations: n, number of COPD patients; %, percentage; SD, standard deviation; COPD, chronic obstructive pulmonary disease; FEV1_ _, forced expiratory volume in first second; CAT score, COPD assessment test score; mMRC Dyspnea Scale, Modified Medical Research Council Dyspnea Scale, ns, non-significant

Variables		Total (n=349)	Group A (n=78)	Group B (n=158)	Group C (n=44)	Group D (n=69)	Significance
Age (years)		60.7(11.2)	59.2(11.3)	59.6(9.8)	61.1(10.1)	64.9(12.1)	^#^p**=0.0069
Age group	<40	10(2.9%)	2(2.6%)	6(3.8%)	1(2.3%)	1(1.4%)	^$^p*=0.0294
	41-50	62(17.8%)	19(24.4%)	27(17.1%)	6(13.6%)	10(14.5%)	
	51-60	112(32.1%)	27(34.6%)	54(34.2%)	15(34.1%)	16(23.2%)	
	61-70	106(30.4%)	17(21.8%)	56(35.4%)	12(27.3%)	21(30.4%)	
	>71	59(16.9%)	13(16.7%)	15(9.5%)	10(22.7%)	21(30.4%)	
Gender	Male	256(73.4%)	57(73.1%)	122(77.2%)	30(68.8%)	48(69.6)	^$^p*=0.509
	Female	93(26.6%)	21(26.9%)	36(22.8%)	14(31.2%)	21(30.4)	
Body mass index(kg/m^2^)		23.4(7.1)	23.1(3.1)	21.9(6.2)	22.8(3.7)	22.4(3.5)	^#^p**=0.0049
Marital status	Married	289(82.8%)	64(82.1%)	132(83.5%)	35(%)	58(%)	^$^p^ns^=0.427
	Unmarried	25(7.2%)	5(6.4%)	15(9.5%)	3(%)	2(%)	
	Others	35(10%)	9(11.5%)	11(6.9%)	6(%)	9(%)	
Residence	Rural	259(74.2%)	54(69.2%)	124(78.5%)	32(72.7%)	49(71%)	^$^p^ns^=0.780
	Urban	58(16.6%)	16(20.5%)	22(13.9%)	8(18.2%)	12(17.4%)	
	Mixed	32(9.2%)	8(10.3%)	12(7.6%)	4(9.1%)	8(11.6%)	
Smoking history	Non-smoker	107(30.7%)	26(33.3%)	48(31%)	15(34.1%)	18(26.1%)	^$^p^ns^=0.755
	Smoker	62(17.8%)	14(17.9%)	32(20.6%)	5(11.4%)	11(15.9%)	
	Ex-smoker	180(51.6%)	38(48.7%)	78(50.4%)	24(54.5%)	40(58%)	
Biomass fuel exposure		69(19.8%)	16(20.5%)	24(15.2%)	15(15%)	14(14.5%)	^#^p^ns^=0.379
Duration of illness (years)		15.4(7.1)	12.9(9.7)	12.6(6.8)	13.6(6.5)	16.2(8)	^#^p**=0.0085
FEV1 (% predicted)		54.6(19.6)	70.6(15.3)	59(15.2)	42.2(15)	36.3(14.5)	^#^p***< 0.0001
CAT		17.7(8.2)	7.4(2.3)	18.4(5.5)	8.4(1.1)	22.7(7)	^#^p***< 0.0001
CAT distribution	No impact, 0-5	18(5.2%)	17(%)	0(%)	1(%)	0(%)	^$^p***< 0.0001
	Low, 6-10	112(32.1%)	61(%)	8(%)	43(%)	0(%)	
	Medium, 11-20	124(35.5%)	0(%)	98(%)	0(%)	26(%)	
	High, 21-30	76(21.2%)	0(%)	47(%)	0(%)	29(%)	
	Very high, 31-40	19(5.4%)	0(%)	5(%)	0(%)	14(%)	
mMRC Dyspnea Scale		2.1(1.0)	0.8(0.4)	2.2(0.4)	0.8(0.4)	2.9(0.8)	^#^p***< 0.0001
mMRC Dyspnea Scale	0	21(6%)	13(17.9%)	0(0%)	8(18.2%)	0(0%)	^$^p***< 0.0001
	1	101(28.9%)	64(82.1%)	0(0%)	36(81.8%)	0(0%)	
	2	152(43.6%)	0(0%)	127(80.4%)	0(0%)	25(36.2%)	
	3	58(16.6%)	0(0%)	30(19%)	0(0%)	28(40.2%)	
	4	17(4.9)	0(0%)	1(0.6%)	0(0%)	16(23.2%)	
Exacerbation history		1(0.9)	0.4(0.5)	0.5(0.5)	2(0.3)	2(0.2)	^#^p***< 0.0001
Exacerbation history	0	135(38.7%)	49(62.8%)	86(54.4%)	0(0%)	0(0%)	^$^p***< 0.0001
	1	101(28.9%)	29(37.2%)	72(45.6%)	0(0%)	0(0%)	
	≥2	113(32.4%)	0(0%)	0(0%)	44(100%)	69(100%)	
Hospitalized due to COPD exacerbation in the last year	0	236(67.6%)	78(100%)	158(100%)	0(0%)	0(0%)	^$^p***< 0.0001
	1	96(27.5%)	0(0%)	0(0%)	38(86.4%)	58(84.1%)	
	≥2	17(4.9%)	0(0%)	0(0%)	6(13.6%)	11(15.9%)	

Comparison of the clinical phenotypes of patients on the basis of symptoms and exacerbation history according to the GOLD 2017 classification

We further classified COPD patients into two groups based on symptoms (less symptomatic (groups A and C) and more symptomatic (groups B and D), Table 4), as well as exacerbation history (low-risk (groups A and B) and high risk (groups C and D), Table 5). The more symptomatic patients (groups B and D) had lower FEV1 (% predicted) and more severe airflow limitation than the less symptomatic patients (groups A and C; 52.1 (18.3) vs. 60.3 (20.5), unpaired t-test with Welch's correction, p=0.0002, Table 4). Similarly, more symptomatic patients had higher values of the CAT (19.7 (6.3) vs. 7.8 (2.1), p< 0.0001, Table 5) and mMRC dyspnea score (2.4 (0.6) vs. 0.8 (0.4), p< 0.0001, Table 4) than the less symptomatic patients. Age, gender, BMI, and illness duration were similar between groups (Table 4). As shown in Table 5, patients in groups C and D who had a high risk of exacerbation were older, had the disease longer, had a higher mMRC dyspnea score and CAT, a lower FEV1, and had worse airflow limitation than patients in groups A and B who had a low risk of exacerbation. 

**Table 4:** Comparison of the clinical phenotype of patients with fewer symptoms (groups A and C) and more symptoms (groups B and D). Notes: Data are expressed in mean (SD), and n (%). ^¥^Unpaired t -test with Welch's correction, ^$^chi-square test, ^#^Fisher's exact test, ^ns^p>0.05, *p≤0.05, **p≤0.01, ***p≤0.001. Abbreviations: n, number of COPD patients; %, percentage; SD, standard deviation; COPD, chronic obstructive pulmonary disease; FEV1, forced expiratory volume in first second; CAT score, COPD assessment test score; mMRC Dyspnea Scale, Modified Medical Research Council Dyspnea Scale; GOLD, Global Initiative for Chronic Obstructive Lung Disease; ns, non-significant.

Variables		Less symptomatic (A+C) group (n=122)	More symptomatic (B+D) group (n=227)	Significance
Age (years)		59.9(10.9)	61.2(10.8)	^¥^p^ns^=0.286
Gender	Male	87(71.3%)	170(74.9%)	^#^p^ns^=0.5244
	Female	35(28.7%)	57(25.1%)	
Body mass index (kg/m^2^)		22.9(3.4)	22.1(5.5)	^¥^p^ns^=0.065
Duration of illness (years)		13(8.6)	13.7(7.3)	^¥^p^ns^=0.425
FEV1 (% predicted)		60.3(20.5)	52.1(18.3)	^¥^p***=0.0002
CAT		7.8(2.1)	19.7(6.3)	^¥^p***< 0.0001
mMRC Dyspnea Scale		0.8(0.4)	2.4(0.6)	^¥^p***< 0.0001
Exacerbation history		1(0.9)	1(0.9)	^¥^p^ns^=0.6355
Hospitalised due to COPD exacerbation in the last year		0.4(0.5)	0.3(0.5)	^¥^p^ns^=0.3223
GOLD grading	1	42(34.4%)	17(7.5%)	^$^p***< 0.0001
	2	33(27.1%)	94(41.4%)	
	3	31(25.4%)	70(30.8%)	
	4	16(13.1%)	44(20.3%)	

**Table 5:** Comparison of the clinical phenotype of patients at low risk (groups A and B) with that of patients at high risk (groups C and D). Notes: Data are expressed in mean (SD), and n (%). ^¥^Unpaired t-test with Welch's correction, ^$^chi-square test,^ #^Fisher's exact test, ^ns^p>0.05, *p≤0.05, **p≤0.01, ***p≤0.001 Abbreviations: n, number of COPD patients; %, percentage; SD, standard deviation; COPD, chronic obstructive pulmonary disease; FEV1, forced expiratory volume in the second; CAT score, COPD assessment test score; mMRC Dyspnea Scale, Modified Medical Research Council Dyspnea Scale; GOLD, Global Initiative for Chronic Obstructive Lung Disease; ns, non-significant

Variables		Low risk (A+B) group (n=236)	High risk (C+D) group (n=113)	Significance
Age (years)		59.5(10.3)	63.4(11.5)	^¥^p**=0.0021
Gender	Male	179(75.9%)	78(69%)	^#^p^ns^=0.194
	Female	57(24.1%)	35(31%)	
Body mass index (kg/m^2^)		22.3(5.4)	22.6(3.6)	^¥^p^ns^=0.5824^¥^
Duration of illness (years)		12.7(7.8)	15.1(7.6)	^¥^P**=0.0081
FEV1 (% predicted)		62.8(16.1)	38.5(15.1)	^¥^p***< 0.0001
CAT		14.8(7)	17.2(8.2)	^¥^P*=0.0135
mMRC Dyspnea Scale		1.8(0.8)	2.1(1.2)	^¥^P**=0.0098
Exacerbation history		0.4(0.5)	2.1(0.2)	^¥^p***< 0.0001
Hospitalised due to COPD exacerbation in the last year		0(0)	1.1(0.3)	-
GOLD grading	1	57(24.2%)	2(1.8%)	^$^p***< 0.0001
	2	110(46.6%)	17(15%)	
	3	60(25.4%)	41(36.3%)	
	4	9(3.8%)	53(46.9%)	

## Discussion

In the current study, 349 stable COPD patients were categorised by using GOLD 2007, GOLD 2011, and GOLD 2017 classification. According to GOLD 2017, 22.4% of patients were in group A, 45.3% in group B, 12.6% in group C, and 19.8% in group D. In groups C and D, illness duration, CAT, mMRC Dyspnea Scale, exacerbation history, and hospitalisation owing to COPD exacerbation were higher than in groups A and B. More symptomatic patients (B and D) exhibited lower FEV1 (% predicted) and more severe airflow limitations. Similar to CAT, more symptomatic patients had a higher mMRC dyspnea score. Groups C and D had older patients, longer disease durations, higher mMRC Dyspnea Scale score and CAT, lower FEV1, and more severe airflow limitation than group B (A and B). These findings of our study demonstrate that patients at risk of exacerbation are identified by the GOLD 2017 classification. Increased mortality risk, lower health status, and decreased lung function are all linked to COPD exacerbations [15-17]. As a result, assessing the risk of an exacerbation should be a part of evaluating stable COPD. Hurst et al. revealed that the best predictor of frequent exacerbations was the history of two or more exacerbations per year [5]. According to the GOLD 2017, a history of exacerbations (two or more exacerbations or one or more exacerbations that required hospitalisation in the previous year) is linked to a higher risk of exacerbations in the future. We observed that groups C and D had higher exacerbations than groups A and B, particularly those that required hospitalisation. These findings suggest that in the COPD population with lower exacerbations, the GOLD 2017 categorisation identifies subjects at risk of moderate-to-severe exacerbations. Our data suggest that patients who have a lower BMI, are more symptomatic, and have major severe airflow restrictions may be more susceptible to exacerbations. These results are consistent with other research showing a link between decreasing airflow limitation and a rising frequency of exacerbations and hospitalisation [15, 18]. Exacerbations may be caused by low BMI and greater symptoms, which have both been identified as potential risk factors [19, 20]. Patients that have this trait may be vulnerable to exacerbations. These results are in line with other studies [11, 21].

Authors demonstrated that all-cause and respiratory mortality were not better predicted by the GOLD 2017 report (basis of symptoms and/or exacerbations) than by the GOLD 2007 or GOLD 2011 classifications [11]. Furthermore, they concluded that the GOLD 2017 report was a superior predictor of all-cause and respiratory mortality than older GOLD reports when utilising a combination of spirometry and symptoms or exacerbations (i.e., a 16 subgroup 1A to 4D) [11]. Here, we also grouped the COPD patients into 16 sub-groups (1A-4D) using GOLD 2017 updates. 

According to GOLD guidelines, any patient with dyspnea, a persistent cough or sputum production, and/or a history of exposure to risk factors for the disease, should be evaluated for COPD. Further, spirometry is necessary to make the diagnosis; the existence of a post-bronchodilator FEV1/FVC less than 0.70 supports the presence of chronic airflow limitation and, consequently, of COPD in patients with exposure to disease symptoms [22]. The primary objective of a COPD evaluation is to identify the degree of airflow restriction, its effects on the patient's overall health, as well as the risk of further events viz. exacerbations, hospital visits or admissions, or even death, or direct selection of the best treatment. Hence, the following elements of the disease must be taken into account independently during COPD assessment in order to fulfil these objectives: whether or not there is a spirometric anomaly and how severe it is, the current nature and symptom severity of the patient, moderate and severe exacerbations in the past and future risk, and existing comorbidities.

Evidence suggests that there is only a weak association between a patient's FEV1, symptoms, and health status impairment [23]. Therefore, a symptomatic evaluation is needed. This symptomatic evaluation is performed by using not only the mMRC Dyspnea scale but also CAT [23-25]. Previously, COPD was mostly characterised by breathlessness, so the mMRC dyspnea scale was used to measure breathlessness for assessment of symptoms, health status, and mortality risk. The mMRC of ≥2 was used as a cutoff to distinguish between ‘less breathlessness’ and 'more breathlessness’ [26]. Later, it was assumed that COPD had consequences other than breathlessness or dyspnea [27]. Due to this, a thorough evaluation of symptoms rather than just a measurement of breathlessness was advised. The Chronic Respiratory Questionnaire (CRQ) [28] and St. George's Respiratory Questionnaire (SGRQ) [14], which are the most comprehensive disease-specific health status questionnaires, are too complex to be used in routine practice, so shorter means, such as the CAT and the COPD Control Questionnaire (CCQ), have been developed. It was suggested that a symptom score of ≥25 on the SGRQ should be used as the cutoff for regular treatment of symptoms like shortness of breath, as different studies provided evidence for treatment recommendations. The CAT cut-off point was set at 10, which is equivalent to SGRQ (≥25) [29]. Exacerbations of COPD are characterised by a sudden worsening of respiratory symptoms that necessitates extra treatment [30]. These incidents are categorised as mild (treated only with short-acting bronchodilators (SABDs)), moderate (treated only with SABDs plus antibiotics and/or oral corticosteroids), or severe (treated alone with SABDs; patient requires visits to the emergency room or hospitalisation) [30]. COPD patients commonly have concomitant chronic illnesses at diagnosis, and COPD is an important component of multi-morbidity development in the elderly due to common risk factors. COPD patients have the same diagnostic, severity evaluation, and care recommendations as other patients.

Thus, the impact of COPD on an individual patient is understood by combining the symptom assessment with the patient's spirometric category and/or risk of exacerbations. Spirometry continues to be essential for the diagnosis, prognosis, and evaluation of other significant therapeutic options and is used for patient symptoms and mild and severe exacerbations. Figure 2 provides an example of this assessment strategy. Patients should undertake spirometry as part of the updated evaluation plan to gauge the severity of airflow limitation (spirometry grade). Additionally, they should have either their symptoms or their dyspnea evaluated using the mMRC or CAT. Finally, a record of their past hospitalizations and mild and severe exacerbations should be made. The alphabets in Groups A to D denote symptom load and risk of exacerbation, which can be utilised for direct therapy, while the numbers (spirometric grade 1 to 4) provide information regarding the severity of airflow limitation.

The thorough and in-depth evaluations of lifestyle, demographic, and clinical characteristics provided are the study's key strengths because they were assessed prospectively from the real-world COPD population from India. The study had a few drawbacks, including the single-centre design, smaller participant pool, exclusion of comorbidities, males constituting the bulk of the participants, and lack of mortality data.

## Conclusions

The present study demonstrates the distribution of COPD patients' clinical phenotypes in an Indian population using the GOLD 2017 classification. Our findings show that the GOLD 2017 classification can identify individuals at risk of exacerbation. Here, a total of 16 sub-groups (1A to 4D) were created using the GOLD 17 report based on a composite of spirometry (to understand the severity of airflow limitation) and symptoms or exacerbations (to understand the severity of symptoms and the risk of exacerbation). Overall, our findings add to the combined COPD assessment in the GOLD 2017 classification to give a more complete picture of the disease.
